# The Mitochondrial Antioxidant SS-31 Modulates Oxidative Stress, Endoplasmic Reticulum Stress, and Autophagy in Type 2 Diabetes

**DOI:** 10.3390/jcm8091322

**Published:** 2019-08-28

**Authors:** Irene Escribano-López, Aranzazu M de Marañon, Francesca Iannantuoni, Sandra López-Domènech, Zaida Abad-Jiménez, Pedro Díaz, Eva Solá, Nadezda Apostolova, Milagros Rocha, Víctor M Víctor

**Affiliations:** 1 Service of Endocrinology, University Hospital Doctor Peset, Foundation for the Promotion of Health and Biomedical Research in the Valencian Region (FISABIO), 46017 Valencia, Spain; 2 CIBERehd—Department of Pharmacology, University of Valencia, 46010 Valencia, Spain; 3 Department of Physiology, University of Valencia, 46010 Valencia, Spain

**Keywords:** Mitochondria, oxidative stress, type 2 diabetes, endoplasmic reticulum stress, autophagy, SS-31

## Abstract

Mitochondrial dysfunction has been shown to play a central role in the pathophysiology of type 2 diabetes (T2D), and mitochondria-targeted agents such as SS-31 are emerging as a promising strategy for its treatment. We aimed to study the effects of SS-31 on leukocytes from T2D patients by evaluating oxidative stress, endoplasmic reticulum (ER) stress and autophagy. Sixty-one T2D patients and 53 controls were included. Anthropometric and analytical measurements were performed. We also assessed reactive oxygen species (ROS) production, calcium content, the expression of ER stress markers GRP78, CHOP, P-eIF2α, and autophagy-related proteins Beclin1, LC3 II/I, and p62 in leukocytes from T2D and control subjects treated or not with SS-31. Furthermore, we have evaluated the action of SS-31 on leukocyte-endothelium interactions. T2D patients exhibited elevated ROS concentration, calcium levels and presence of ER markers (*GRP78* and *CHOP* gene expression, and GRP78 and P-eIF2α protein expression), all of which were reduced by SS-31 treatment. SS-31 also led to a drop in *BECN1* gene expression, and Beclin1 and LC3 II/I protein expression in T2D patients. In contrast, the T2D group displayed reduced p62 protein levels that were restored by SS-31. SS-20 (with non-antioxidant activity) did not change any analyzed parameter. In addition, SS-31 decreased rolling flux and leukocyte adhesion, and increased rolling velocity in T2D patients. Our findings suggest that SS-31 exerts potentially beneficial effects on leukocytes of T2D patients modulating oxidative stress and autophagy, and ameliorating ER stress.

## 1. Introduction

Type 2 diabetes (T2D) represents a serious global problem with a worryingly high rate worldwide, constituting one of the main public health challenges of the 21st century. T2D is a metabolic disruption characterized by insulin resistance (IR) and β cell failure. In those affected, the persistent exposure to a hyperglycemic environment promotes excessive generation of reactive oxygen species (ROS) and it leads to the imbalance of antioxidant defenses [1], inducing oxidative stress, which contributes to IR and the activation of pro-inflammatory signaling pathways [2], both thought to play key roles in the complications associated with T2D.

Oxidative stress and endoplasmic reticulum (ER) stress are closely linked. Indeed, an altered redox balance has a major impact on ER folding capacity. Under pathological conditions such as T2D, ER homeostasis is disturbed due to an accumulation of misfolded proteins [3,4], in response to which the unfolded protein response (UPR) is activated in order to (i) upregulate the expression of chaperones and aid the folding of ER proteins (ii) and degradation of proteins, and (iii) to prevent protein synthesis [5,6]. It is known that antioxidant production is one of the restorative functions of the UPR, which coordinates the activation of the trans-membrane ER resident protein (PERK) signaling pathway, thus allowing the cell to adapt to oxidative and ER stress [7,8]. The ER stress response also includes mechanisms of autophagy induction, and it has been demonstrated that low-grade autophagy reduces ER stress by destruction of the ubiquitinated unfolded/misfolded dysfunctional proteins and damaged organelles that result from said stress [9]. The onset of autophagy involves the formation of an autophagosome, a process in which several autophagy-related genes coordinate to engulf the defective material in a double membrane. This process is initiated when the complex formed by Beclin1/Vps34/VPs15/UVRAG—known as PI3K complex III—nucleates the formation of the autophagosome. In parallel, the cytosolic protein microtubule-associated to 1B-light chain 3 (LC3 I) is conjugated to a phosphatidylethanolamine to form LC3 II. In this form, LC3 II migrates to the growing autophagosome and helps to build the double membrane. The ubiquitinated protein and defective organelles are detected by sequestosome 1 (SQSTM1)—also known as the p62 protein—which associates itself to membrane-bound LC3 II. The autophagosome then fuses with a lysosome, which pours its hydrolytic enzymes into the inner space of the autophagosome, thereby degrading its content [10,11]. This is usually a rescue mechanism in situations of stress. However, when ER stress is prolonged, the autophagy activated as a result can lead to severe cell damage and, eventually, to apoptosis [12,13]. Incipient insult has serious consequences for the balance of pro- and anti-survival signals. Therefore, the mechanisms of oxidative stress, ER stress and autophagy are closely related to each other and are considered key targets for understanding the development of T2D. In the present work, we have studied these processes by analyzing general markers for their activation.

Mitochondria are essential to the control of cellular homeostasis, cell death and apoptosis. Furthermore, overproduction of ROS occurs mainly in mitochondria, through the electron transport chain [14,15], thus attributing these organelles a key role in the development and control of metabolic diseases such as T2D. For the aforementioned reasons, the identification of novel mitoprotective therapies may lead to the prevention and successful treatment of the complications associated with this disease. 

One of the molecules that might be beneficial in mitochondria-based diseases is the mitochondria-targeted antioxidant SS-31 (D-Arg-2’6’-dimethylTyr-Lys-Phe-NH_2_), a member of the Szeto–Schiller (SS) peptide family, aromatic-cationic tetrapeptides targeted to cardiolipin on the inner mitochondrial membrane via hydrophobic and electrostatic interactions. There, they increase ATP production, thus restoring cellular function and preserving vital ATP-dependent processes [16,17,18]. Their antioxidant action is due to two actions, the dimethyltyrosine residue, scavenging H_2_O_2_ and ONOO- and inhibiting lipid peroxidation. In addition, preclinical studies support their potential use in neurodegenerative disorders and ischaemia-reperfusion injury [19]. Our group has already demonstrated that SS-31 increases SIRT1 levels in leukocytes and ameliorates inflammation, oxidative stress and leukocyte-endothelium interactions in T2D [20].

In the present study, we set out to explore the effects of SS-31 on leukocytes of T2D patients by evaluating different key pathways including oxidative stress, ER stress and autophagy. We have used peripheral blood leukocytes as surrogate model for the general/systemic oxidative stress and its consequences present in T2D. Actually, T2D has been widely related with leukocyte dysfunction [21,22,23,24,25]. Peripheral blood leukocytes are the primary sensors of the alterations in the presence of different soluble molecules in the bloodstream [26,27,28,29,30]. More precisely, we have employed polymorphonuclear cells (PMNs) which present higher vulnerability to oxidative damage in T2D compared to mononuclear cells [21]. Numerous studies suggest that the continuous exposure of leukocytes to high circulating levels of glucose, lipids, insulin, and proinflammatory cytokines (known as the T2D environment) alters the cell metabolism and affect the cell ability to manage stress situations. These alterations have a direct impact on the leukocytes’ function and main pathways such as oxidative stress regulation, ER stress, autophagy, and mitochondrial homeostasis [31,32,33,34,35]. Previous research stated that different molecules, drugs or antioxidants can relieve the stress response [36,37,38,39]. Taken into account these facts, we consider that PMNs are a readily available, representative and valid model to evaluate the influence of ROS on the different pathways related to T2D [21].

## 2. Experimental Section

### 2.1. Human Subjects

A total of 114 subjects were included in the study population, specifically 61 T2D patients and 53 healthy controls recruited from the Service of Endocrinology and Nutrition of University Hospital Doctor Peset (Valencia, Spain) and adjusted for age and sex. All subjects gave their written informed consent to participate in the study and the protocols followed were approved by our hospital’s Ethics Committee for Clinical Investigation (ID: 97/16), in line with the ethical principles of the Helsinki declaration. All T2D patients in this study have suffered from T2D for at least 5 years, which ensures that they display a chronic phenotype.

The American Diabetes Association’s criteria were used for T2D diagnosis, and exclusion criteria were history of cardiovascular disease (CVD), presence of morbid obesity or autoimmune, hematological, malignant, infectious, organic, or inflammatory disease, and insulin treatment.

### 2.2. Sample Collection

Venous blood samples were taken from the antecubital vein and collected in Vacutainer^®^ tubes in fasting conditions, between 8 AM and 10 AM. Anthropometric parameters—weight (kg), height (m), body mass index (BMI; kg/m^2^), systolic and diastolic blood pressure (SBP/DBP; mmHg), and waist circumference (cm)—were assessed.

### 2.3. Laboratory Tests

Fresh blood samples were centrifuged for 10 min at 1500 g at a temperature of 4 °C in order to separate serum from the blood. Serum levels of fasting glucose, total cholesterol and triglycerides were determined by enzymatic method, high-density lipoprotein cholesterol (HDL-c) was recorded employing a Beckman LX-20 autoanalyzer (Beckman Coulter, La Brea, CA, USA) using a direct method, and low-density lipoprotein cholesterol (LDL-c) content was quantified with Friedewald’s formula. Insulin levels were obtained by an immunochemiluminescence assay, and HOMA-IR index (fasting insulin (µU/mL) × fasting glucose (mg/dL)/405) was calculated to estimate IR. The percentage of glycated hemoglobin (HbA1c) was determined with an automatic glycohemoglobin analyzer (Arkray, Inc., Kyoto, Japan) and an immunonephelometric assay was used to measure high-sensitive C-reactive protein (hs-CRP) levels.

### 2.4. Leukocyte Isolation

Human polymorphonuclear leukocytes (PMNs) were isolated from heparinized blood samples incubated for 45 min with 1:2 volumes of dextran solution (3% in NaCl 0.9%; Sigma Aldrich, MO, USA). The supernatant was centrifuged over Fycoll-Hypaque (GE Healthcare, Uppsala, Sweden) at 650 g for 25 min and the pellet lysed to remove the remaining erythrocytes. It was then incubated with lysis buffer (5 min at room temperature) and centrifuged at 240 g. Pellets containing leukocytes were then washed twice and resuspended in Hank’s balanced salt solution (HBSS; Sigma Aldrich, MO, USA). A Scepter 2.0 device (Millipore Iberica, Madrid, Spain) was employed for the cell count. The cellular suspension was split into two samples, which were treated under the same conditions with concentrations that did not affect the cells’ viability; one was incubated with SS-31 (100 nM, 30 min), and the other with SS-20 (100 nm, 30 min, without antioxidant activity).

### 2.5. PMN-Endothelium Interaction Assay

PMNs were isolated as previously described by our group [20]. In this assay, we employed a 1.2 mL aliquot of PMNs obtained from the peripheral blood of control and T2D subjects with a density of 10^6^ cells/mL in complete RPMI. Prior to this, primary cultures of human umbilical cord endothelial cells (HUVEC) were established. HUVEC were isolated as previously reported [20]. On the day of the experiment, the PMN aliquot was passed through the endothelial monolayer at a speed of 0.3 mL/min during a 5-min period, which was recorded. Next, the number of rolling PMNs, as well as their velocity and adhesion to the endothelial monolayer were recorded. The number of rolling PMNs was measured as those rolling for 1 min (recorded on video). Velocity was assessed by determining the time in which 15 rolling PMNs covered a distance of 100 micrometers. Adhesion was analyzed by counting the number of PMNs adhering to the endothelium for at least 30 s in 5 fields.

### 2.6. Quantitative Fluorescence Microscopy

Fluorescence probes 2′,7′-dichlorodihydrofluorescein diacetate (DCFH-DA; 5 × 10^−6^ mol/L), MitoSOX (5 × 10^−6^ mol/L) and (acetyloxy)methyl ester (Fluo-4 AM; 1 × 10^−6^ mol/L) were used to assess total ROS, mitochondrial ROS and calcium levels, respectively. DCFH-DA is routinely used in intact cells, being taken up and deacetylated by endogenous hydrolases to a form (DCFH) that is then oxidized by peroxides to fluorescent 2′,7′-Dichlorofluorescein (DCF). MitoSOX, a mitochondria-targeted dihydroethidium (by addition of a triphenylphosphonium group) is a probe widely used to detect superoxide. To perform these assays, isolated leukocytes were placed in 48-well plates and incubated for 30 min at 37 °C with the appropriate fluorochrome, diluted in phosphate-buffered saline (PBS; Sigma Aldrich, MO, USA). Fluorescence intensity was then recorded with a fluorescence microscope (IX81; Olympus Corporation, Shinjuku-ku, Tokyo, Japan) coupled to the static cytometry software “ScanR” (Olympus). Fluorescence units of these measurements were normalized with respect to the control group, in which the mean fluorescence units were considered 100%, and the data were relativized to that fluorescence value. Experiments were performed in duplicate and 16 images per well were obtained and analyzed obtaining a mean fluorescence value. The mean value of these two replicates of each sample was used for data representation and statistical analysis. Nuclei were detected with Hoechst 33342. All fluorochromes were supplied by Thermo Fisher Scientific, Waltham, MA, USA.

### 2.7. Flow Cytometry

ROS generation in human leukocytes was analyzed using whole blood by flow cytometry using DCFH-DA (5 × 10^−6^ mol/L) as marker dye. The distribution of different leukocyte subsets was analyzed in peripheral blood using a single staining (CD45), no-lyse no-wash method. CD45 positive cells (marked with the fluorescent probe APC Mouse Anti-Human CD45, BD Biosciences, San Jose, CA, USA) and the morphological characteristics of the cells (FSC and SSC parameters) were used for determining the PMNs cellular subset as shown in previous work [40,41]. Briefly, 200 µL of heparinized blood were incubated with 4 µL of CD45 monoclonal antibody for 20 min at room temperature in darkness, in the presence and absence of several treatments. For this assay, 500 µL of stained blood diluted 20-fold in PBS was incubated for 30 min at 37 °C with the fluorochrome DCFH-DA. Samples were acquired for 10,000 individual cells by BD Accuri^TM^ C6 Plus Flow Cytometer (BD Biosciences, San Jose, CA, USA) and ROS production was quantified by median fluorescence intensities.

### 2.8. Western Blotting (WB)

Leukocyte pellets were homogenized and incubated on ice in cell lysis buffer for 15 min (10 mM HEPES pH 7.5, 10 mM NaCl, 2 mM MgCl_2_, 1 mM EDTA, 1 mM EGTA, 0.5% Nonidet P-40, 1 mM DTT, ‘Complete Mini’ and ‘Pefabloc’ protease inhibitor cocktail from Roche Diagnostics and phosphatase inhibitor mixture: 10 mM NaF and 0.1 mM Na_3_VO_4_); tubes were vortexed to disrupt the cell membranes and centrifuged at 4 °C for 10 min. The supernatants were stored at −70 °C till further use as cytoplasmic extracts. The pelleted nuclei were resuspended in the nuclear extraction buffer (25 mM HEPES pH 7.5, 500 mM NaCl, 9 % glycerol (v/v), 5 mM MgCl_2_, 0.5 % Nonidet P-40, 1 mM DTT) supplemented with protease inhibitors (‘Complete Mini’ protease inhibitor cocktail, and ‘Pefabloc’, both from Roche Diagnostics) and 10 mM NaF as a phosphatase inhibitor, and were incubated on ice for 10 min under sonication. Nuclear extracts were collected by centrifugation for 10 min at 4 °C, and were either immediately used or stored at −70 °C. Protein concentration was determined with a BCA protein assay kit (Thermo Fisher Scientific, Waltham, MA, USA). Next, 25 µg proteins per sample were loaded onto SDS-polyacrilamide gels. Gel electrophoresis was performed at 120 V, 90 min, followed by transfer to nitrocellulose membranes (Bio-Rad, Hercules, CA, USA) at 400 mA, for 1 h. After blocking at RT for 1 h in 5% non-fat milk in TBST buffer containing 25 mM Tris, 150 mM sodium chloride and 0.1% Tween20, at pH 7.5, membranes were incubated overnight at 4 °C with anti-glucose-regulated protein 78 kDa (GRP78) rabbit polyclonal antibody (Abcam, Cambridge, UK), anti-phosphorylated eukaryotic translation initiation factor 2, subunit 1 alpha (eIF2α-pS52) rabbit polyclonal antibody (Life Technologies, Carlsbad, CA USA), anti-Beclin1 (BECN1) rabbit polyclonal antibody (Abcam, Cambridge, UK), anti-light chain 3 (LC3) rabbit polyclonal antibody (Millipore Iberica, Madrid, Spain), anti-sequestosome 1 (SQSTM1/p62) mouse monoclonal antibody (Abnova, Taipei, Taiwan) or anti-Actin rabbit polyclonal antibody (Sigma Aldrich, St Louis, MO, USA), followed by horseradish peroxidase (HRP) goat anti-rabbit (Millipore Iberica, Madrid, Spain) or HRP goat anti-mouse (Thermo Fisher Scientific, Waltham, MA, USA) secondary antibodies as appropriate, for 1 h at RT. Protein expression was assessed with ECL plus reagent (GE Healthcare, Amersham Place, Litte Chalfont, UK) or Supersignal West Femto (Thermo Fisher Scientific, Waltham, MA, USA). A Fusion FX5 acquisition system (Vilbert Lourmat, Marne La Vallée, France) was employed for chemiluminescence signal detection, which was analyzed by densitometry using Bio1D software (Vilbert Lourmat, Marne La Vallée, France). For quantification of the expression level of the studied protein, an internal control was included in each blot and the expression was normalized to that of actin in the same sample.

### 2.9. Quantitative RT-PCR (qRT-PCR)

Total RNA was isolated from leukocytes with the GeneAll^®^ Ribospin^TM^ kit following the manufacturer’s instructions (GeneAll Biotechnology, Hilden, Germany). RNA concentrations were measured using Nanodrop 2000c (Thermo Fisher Scientific, Waltham, MA, USA), and 1 µg of the extracted RNA was employed in the following steps. To detect the expression of genes involved in autophagy and ER stress, the RevertAid First Strand c-DNA Synthesis kit (Thermo Fisher Scientific) and KAPA SYBR FAST universal master mix (Applied Biosystems-Thermo Fisher Scientific, Walthman, MA, USA ) were used. RT-qPCR analysis was performed with a 7500 Fast real-time PCR system (Life Technologies, Carlsbad, CA, USA) (Table 1). Fold changes were calculated by the 2^−ΔΔCt^ method through Expression Suite software (Life Technologies) and relative gene expression of *GRP78, DDIT3/CHOP* (CCAAT/enhancer-binding protein (C/EBP) homologous protein), *BECN1* and *SQSTM/p62* was calculated using *GAPDH* as a housekeeping control.

### 2.10. Statistical Analysis

All data were analyzed with SPSS 17.0 software (SPSS Statistics Inc., Chicago, IL, USA). Values are expressed as mean and standard deviation (SD) for parametric data; while the median (25th–75th percentiles) is presented for non-parametric data. Bar graphs show mean and standard error of the mean (SEM) in the figures.

In the case of the variables with normally distributed data, groups were compared with a Student’s *t*-test, while a Mann–Whitney *U* test was employed for non-normally distributed ones, and the chi-square test for proportion of frequencies. To examine the main effects of the treatment, the study groups were compared with one-way analysis of variance (ANOVA) followed by a Newman–Keuls post hoc test. In addition, the prominent influence of BMI was reduced by means of an analysis of covariance with a univariate general linear model. Differences were considered to be significant when *p* < 0.05, applying a confidence interval of 95% in every comparison. Graphs were plotted with GraphPad Prism 4.0 (GraphPad, La Jolla, CA, USA).

## 3. Results

### 3.1. Clinical and Endocrine Parameters

Our study was carried out in a population of 53 healthy volunteers (mean age 51.7 ± 9.3) and 61 T2D patients (mean age 55.1 ± 10.2), both of which groups had a similar gender distribution. The results of the anthropometric and analytical evaluations are shown in Table 2. As expected, an altered carbohydrate metabolism was observed in T2D patients in comparison with the control group, with glucose, HOMA-IR and HbA1c being significantly higher (*p* < 0.001). Moreover, the T2D group showed higher values for upper waist circumference (*p* < 0.01), SBP, weight, BMI, insulin and hs-CRP levels (*p* < 0.001) than control subjects. Regarding lipid profile, a higher triglyceride concentration (*p* < 0.01) and lower HDL-c (*p* < 0.001) were characteristics of the T2D patients. However, due to lipid-lowering medication received, total cholesterol and LDL-c levels were lower in the diabetic group than in healthy controls (*p* < 0.001) (56.9% were taking statins, 10.3% fibrates, and 3.4% ezetimibe). Given that BMI was significantly different in T2D patients, data were adjusted for this variable, but statistical differences remained similar.

### 3.2. Leukocyte Function

For assessing the influence of T2D and SS-31 treatment on one of the main functions of PMN, which is interaction with the endothelial monolayer, we performed a parallel plate flux chamber assay. As stated in methods, PMN were perfused through a monolayer of confluent endothelial cells for assessing those interactions. As shown in Figure S1, T2D enhanced the flux of leukocytes (Figure S1A), reduced its velocity (Figure S1B) which allowed them to adhere more to the endothelial monolayer (Figure S1C). When treated with SS-31, those interactions were significantly reduced. This result shows that leukocyte function is positively affected by SS-31 in T2D PMN. SS-20 did not alter those parameters in any of the analyzed samples.

### 3.3. Oxidative Stress: ROS Production

**Figure 1:**
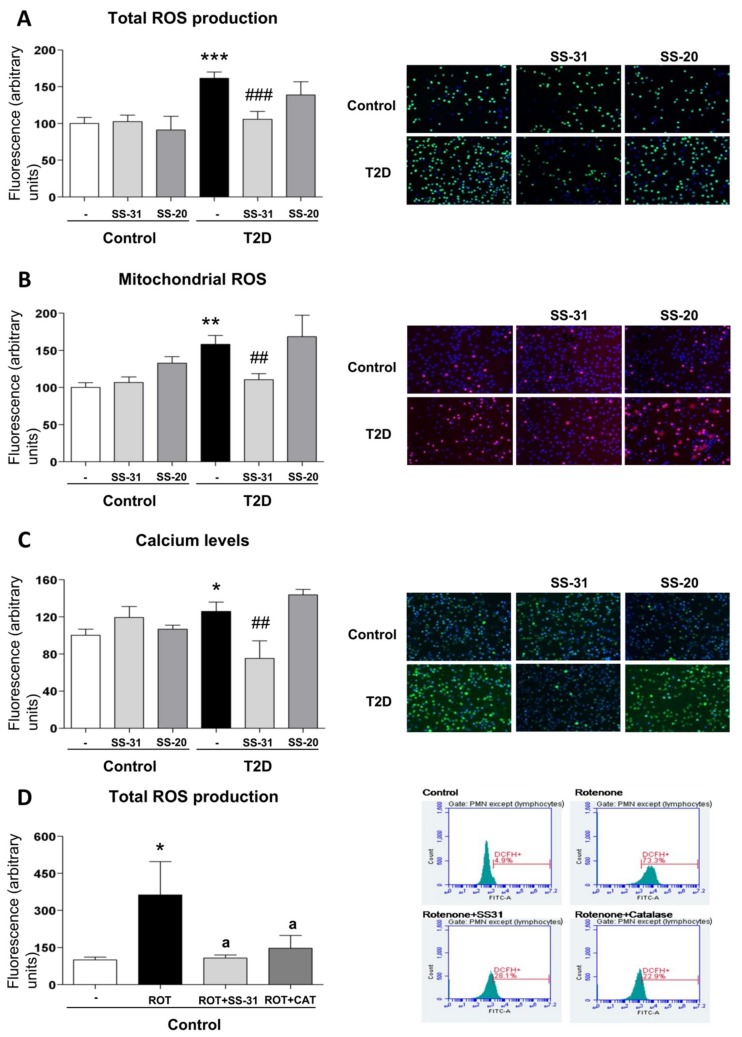
Effects of Szeto–Schiller (SS)-31 (30 min, 100 nM) on total and mitochondrial ROS production, and calcium levels in leukocytes from type 2 diabetes (T2D) patients and healthy subjects. (**A**) reactive oxygen species (ROS) production, measured as deacetylated by endogenous hydrolases to a form (DCFH)-DA fluorescence. (**B**) Mitochondrial ROS production, assessed as MitoSOX fluorescence. (**C**) Calcium levels, determined as Fluo-4 fluorescence. Representative fluorescence microscopy images are also shown. (**D**) Analysis of total ROS levels, measured as DCFH-DA fluorescence in leukocytes from healthy subjects upon a positive control treatment (rotenone, ROT) in the presence or absence of SS-31 or catalase (CAT) and representative cytograms of the 4 groups stained with APC-CD45 and DCFH-DA. 10,000 cells were analyzed in each experiment. *n* = 6. * *p* < 0.05, ** *p* < 0.01 and *** *p* < 0.001 with regard to control group; ^##^ *p* < 0.01 ^###^ *p* < 0.001 vs. non-treated T2D group; ^a^ *p* < 0.05 vs. rotenone treatment.

Total (DCFH-DA fluorescence) and mitochondrial (MitoSOX fluorescence) ROS were considerably increased in leukocytes of T2D patients in comparison with control subjects (Figure 1A,B; *p* < 0.001), and these effects were reversed by SS-31 (Figure 1A,B; *p* < 0.001, *p* < 0.01 respectively) in leukocytes of T2D patients, while no differences were observed in controls. The SS-20 compound did not alter these oxidative stress parameters. The specificity of the observed response was corroborated by cytometry analysis of the effect of a positive control, rotenone, a well-known inhibitor of Complex I of the electron transport chain whose action induces mitochondrial superoxide production [42]. Incubation with whole blood from control subjects with rotenone (50 µM, 20 min) led to a major increase in total cellular ROS (detected by DCFH-DA) and this effect was reversed with the treatment of both SS-31 and catalase (Figure 1D; *p* < 0.05). Thus, our data show that SS-31 exerts an antioxidant action by reducing total and mitochondrial ROS production.

### 3.4. Calcium Levels

In the T2D study population, intracellular calcium content—measured as Fluo4-AM fluorescence—was higher than in the control group (Figure 1C; *p* < 0.05), while under treatment with SS-31, calcium levels in T2D patients reached similar values to those in healthy subjects (Figure 1C; *p* < 0.01). The marked decrease of calcium content in the SS-31-treated T2D group in comparison with healthy volunteers may indicate an attenuation of ER stress in these patients given the fact that ER stress is often related to an increase in cytosolic calcium content SS-20 treatment did not modify calcium content in any condition.

### 3.5. Regulation of UPR Signalling

**Figure 2:**
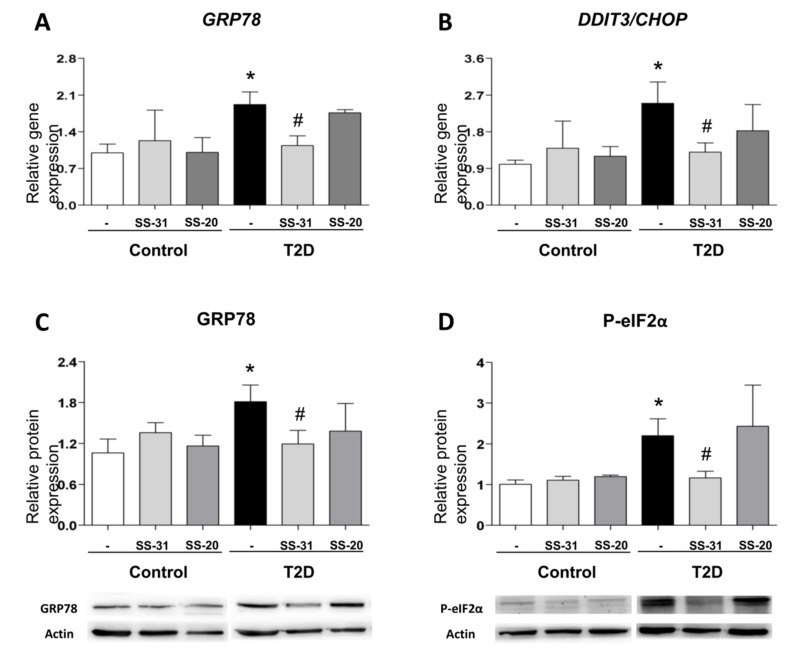
Evaluation of endoplasmic reticulum (ER) stress parameters in leukocytes from T2D patients and controls in the absence and presence of SS-31 (30 min, 100 nM) (**A**) *GRP78* expression. (**B**) *DDIT3/CHOP* expression. (**C**) GRP78 protein levels and representative western blotting (WB) images. (**D**) P-eIF2α protein levels and representative WB images. * *p* < 0.05 with regard to control group ^#^ *p* < 0.05 vs. non-treated T2D group.

ER stress markers were determined in order to analyze UPR activation in leukocytes from T2D patients and control subjects. A higher peak in *GRP78* expression was observed in the T2D vs. control group (Figure 2A; *p* < 0.05); similarly, *DDIT3*/*CHOP* expression was augmented in T2D patients (Figure 2B; *p* < 0.05). Interestingly, SS-31 treatment reduced mRNA levels of both genes in leukocytes from T2D patients (Figure 2A,B; *p* < 0.05). Furthermore, the treatment with the mitochondria-targeted antioxidant SS-31 had no effect about protein levels of GRP78 and P-eIF2α on leukocytes of control subjects (Figure 2C,D) while a reduction in these ER stress parameters was observed in leukocytes from T2D patients with T2D (Figure 2C,D; *p* < 0.05). None of these markers were altered by treatment with SS-20.

These findings suggest that SS-31 can attenuate ER stress in the leukocytes of T2D patients.

### 3.6. Autophagy Assessment

**Figure 3:**
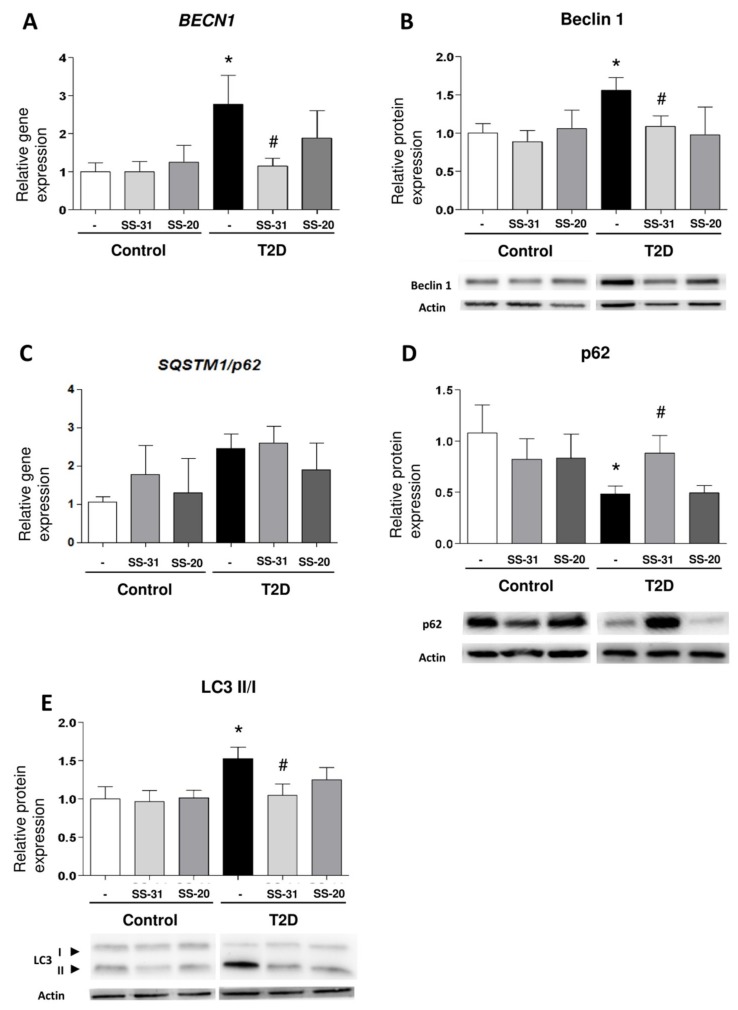
Study of autophagy-related parameters in leukocytes from control subjects and T2D patients in the presence and absence of SS-31 (30 min, 100 nM). (**A**) *BECN1* expression. (**B**) Beclin 1 protein expression and representative WB images. (**C**) *SQSTM1/p62* expression (**D**) p62 protein expression and representative WB images (**E**) LC3 II/I ratio of protein expression and representative WB images. * *p* < 0.05 with regard to control group, ^#^ *p* < 0.05 vs. non-treated T2D group.

*BECN1* gene expression were enhanced in leukocytes from T2D patients with respect to those of healthy controls (Figure 3A; *p* < 0.05), a trend that was reversed by treatment with SS-31 (Figure 3A; *p* < 0.05). In leukocytes from T2D patients treated with SS-31, this trend was also accompanied by a significant reduction of protein expression of distinct markers of autophagy such as Beclin1 and the ratio of LC3 II/I (Figure 3B,C; *p* < 0.05). p62 protein level was significantly lower in leukocytes from diabetic patients compared to controls, however its mRNA levels were more abundant in T2M patients which is indicative of enhanced autophagy. Treatment of leukocytes from T2D patients with SS-31 reversed the protein level of p62 (Figure 3D; *p* < 0.05), while no changes were seen in the gene expression of *SQSTM1/p62* suggesting that SS-31 can modify autophagy at protein level. On the other hand, no significant differences were observed in control group or with SS-20 treatment.

These results provide some evidence that the mitochondria-targeted antioxidant SS-31 reduces parameters of autophagy in leukocytes from T2D patients.

### 3.7. Analysis of Pharmacologically Induced ER Stress and Autophagy

**Figure 4:**
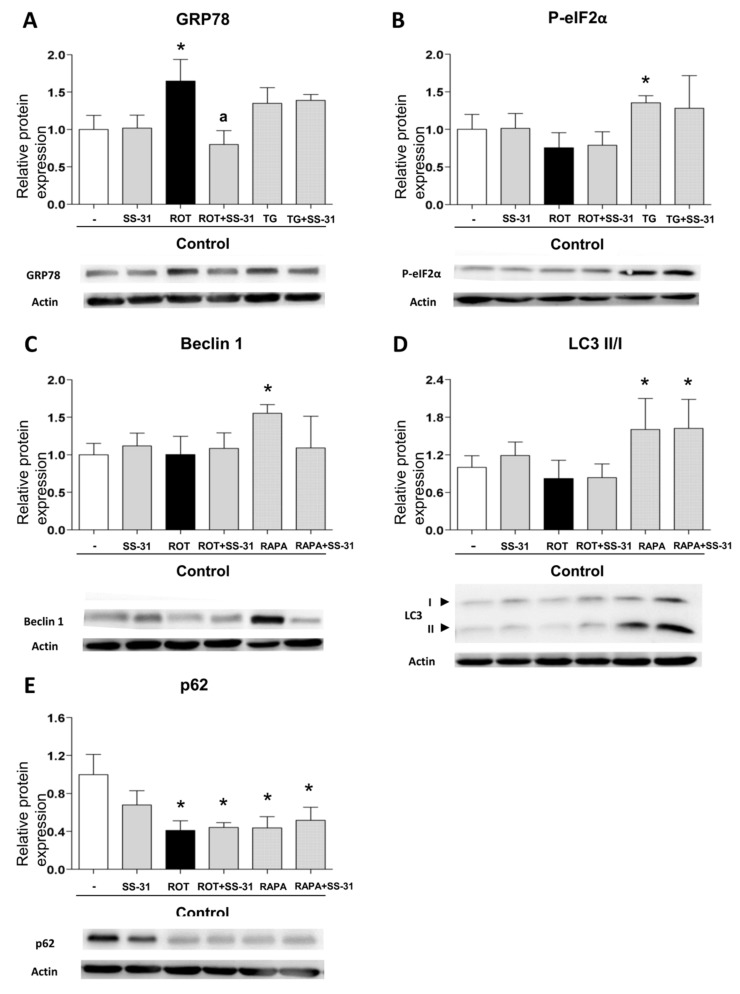
Study of the expression of protein markers of ER stress and autophagy, induced pharmacologically in leukocytes from healthy controls, in the presence and absence of SS-31 (30 min, 100 nM). (**A**) GRP78, (**B**) P-eIF2α, (**C**) Beclin1, (**D**) LC3 II/I ratio, and (**E**) p62. Representative WB images are also shown. * *p* < 0.05 with regard to control group; ^a^ *p* < 0.05 vs. rotenone-treated group. *n* = 6. ROT, rotenone (50 µM, 20 min); TG, thapsigargin (1 µM, 20 min); RAPA, rapamycin (0.5 µM, 30 min).

Given that leukocytes from T2D display markers of ER stress and activated autophagy, and the observation that both effects can be alleviated with SS-31 treatment, we set to explore the connection between these processes. For this, we evaluated the capacity of SS-31 to interfere with pharmacologically induced ER stress (thapsigargin) and autophagy (rapamycin). Treatment with thapsigargin (1 µM, 20 min) produced a significant increase in the protein content of P-eIF2α and a slight increase in GRP78, however these effects were not impaired if cells were co-treated with SS-31 (Figure 4A,B). The sesquiterpene alkaloid thapsigargin, a highly selective inhibitor of sarcoplasmic/endoplasmic reticulum Ca^2+^ ATPase (SERCA) prevents Ca^2+^ transport into the ER lumen, which leads to its subsequent increase in the cytosol, and promotes accumulation of unfolded proteins and perturbation of intracellular Ca^2+^ homeostasis [42]. On the contrary, SS-31 was able to prevent the increase in GRP78 protein content when it was induced by the mitochondrial inhibitor rotenone (Figure 4A), a finding that reinforces the ability of SS-31 to act as an antioxidant. Regarding autophagy, as expected, leukocytes from healthy subjects exposed to the pharmacological inducer rapamycin (0.5 µM, 30 min) displayed enhanced autophagy as evidenced by the incremented Beclin1 and LC3 II levels (Figure 4C,D), and the diminished p62 protein content (Figure 4E). Rapamycin inhibits the mTOR complex, a central negative regulator of autophagy in the mammalian cell, thus triggering a strong autophagic response [42,43]. Importantly, SS-31 treatment had no effect on these alterations (Figure 4C–E), a finding that once more underscores the specificity of SS-31 action in the complex metabolic disturbances in leukocytes of T2D patients. We also evaluated autophagy induction in the cells exposed to rotenone and observed no increase in Beclin1 and LC3 II levels. The protein levels of p62 were diminished; however, given the lack of changes in the LC3 II/I ratio the effect may be evidence of an autophagy-independent regulation.

## 4. Discussion

Based on renewed concepts of T2D pathogenesis, the targets of a potential therapy for this chronic progressive disease include, not only glucose homeostasis correction, but also modulation of cellular stress and mitochondrial function in highly metabolic tissues, with the aim of attenuating insulin resistance and low-grade inflammation and ameliorating β cell function and mass, thus preventing the development of macro- and microvascular complications.

In this sense, the mitochondrial antioxidant SS-31 has been described to exert protective effects in several disease models. Indeed, SS-31 has previously been reported to attenuate renal injury in diabetic nephropathy through an antioxidant effect [44]. Furthermore, Zhu et al. have demonstrated that SS-31 attenuates the severity of lung damage by modulating mitochondrial dysfunction in a mouse model of spinal cord injury [45]. However, the exact pathophysiological mechanism involved in the protective effects of SS-31 on leukocytes in T2D is not fully understood. For this reason, the present study was designed to evaluate whether SS-31 can modulate oxidative stress, ER stress and autophagy in leukocytes of T2D patients, three important pathways involved in the development of T2D.

The pathophysiology of T2D is associated with an impairment of β cell function and, consequently, IR, a hallmark of this disease [46]. Nevertheless, whether cell failure is a primary cause of T2D or secondary to associated long-term metabolic abnormalities is yet to be confirmed, though increased oxidative stress, ER stress and autophagy are thought to be involved [47]. In fact, previous studies have suggested that alterations in ΔΨ_m_ disturb mitochondrial dynamics, eventually promoting a failure in glucose-stimulated insulin secretion [48]. Moreover, our group has previously demonstrated oxidative stress and mitochondrial dysfunction in leukocytes from T2D patients [49]. In this sense, SS-peptides can scavenge ROS, and these molecules have been shown to exert beneficial effects against mitochondrial dysfunction [19,50]. SS-31 protects mitochondria against oxidative damage by accumulating in the inner mitochondrial membrane, a location close to the site of ROS production. In fact, after crossing the mitochondrial outer membrane, SS-31 associates with cardiolipin, an anionic phospholipid expressed exclusively in the inner mitochondrial membrane. Furthermore, SS-31 seems to protect cristae architecture by alleviating mitochondrial oxidative stress and preventing cytochrome c peroxidase activity [17,19].

In the present study, we have found that the leukocytes of T2D patients have functional alterations compared to those of control individuals, as shown in Figure S1. SS-31 is able to rescue the parameters of leucocyte–endothelial interactions which confirms that SS-31 can modulate leukocyte function. The fact that the effects can be ameliorated with SS-31 but not with SS-20 shows that the alterations of the leukocyte function in T2D leukocytes can be due to the high levels of total and mitochondrial ROS levels compared to controls. Fluorescent probes are widely used for ROS detection in biological systems; DCFH–DA has been suggested as a relatively specific probe for H_2_O_2_, while dihydroethidium seems to be more suitable for superoxide. However, abundant evidence over the past years has shown that all fluorescent probes for ROS detection suffer a lack of selectivity given that they react with various types of ROS, and therefore in living cells or tissues they are generally used for detecting total oxidative activity. In order to reaffirm our findings, we have employed two fluorescent probes and verified the specificity of the detection by studying a positive control of mitochondrial ROS generation, rotenone.

One of the leading hypotheses regarding the onset of IR is that enhanced ROS production triggers ER stress, which leads to activation of the UPR. In relation to this, ER stress is considered a target mechanism under IR conditions. An association between IR and mitochondrial abnormalities, such as lower numbers of mitochondria, reduction in mitochondrial oxidative enzyme activity or mitochondrial dysfunction, have been reported in human studies [51,52]. Furthermore, it has been described that ER stress is related to apoptosis in leukocytes from T2D patients [53]. In addition, a study by Sage et al. demonstrated that levels of ER stress markers such as GRP78, sXBP1 and CHOP correlated positively with glucose levels in leukocytes from patients with metabolic syndrome [54]. In line with such data, we have shown in previous studies that leukocytes from T2D patients exhibit increased ER stress markers which display enhanced GRP78, P-eIF2α and ATF6 protein levels [35]. Interestingly, the present study shows that SS-31 treatment in leukocytes from T2D patients reduces GRP78 and P-eIF2α protein levels, and *GRP78* and *CHOP* mRNA levels, suggesting that this molecule could promote the restoration of cell homeostasis to battle ER stress. The reduction of intracellular calcium levels under treatment with SS-31 described in the present study could also be indicative of lower ER stress compared to untreated T2D leukocytes, but these data need to be considered with caution given that with our methodology we cannot determine the subcellular source of increased calcium. This idea is further enforced by the fact that SS-31 did not alleviate ER stress triggered by other types of stimuli such as thapsigargin, an ER stressor with a direct effect on ER calcium homeostasis.

ER stress can also induce autophagy, and in this sense Gonzalez et al. have described that cleavage and lipidation of microtubule-associated protein LC3 I into LC3 II is mediated by the phosphorylation of PERK/eIF2α [55]. Importantly, we have previously demonstrated in leukocytes from T2D patients that UPR activation occurs in parallel with autophagy [35]. The present study describes an increase in Beclin1 and LC3-II levels in T2D patients compared to controls which is indicative of increased generation of autophagosomes. As this occurs concomitantly with a decrease in p62 protein levels, we believe that it may suggest an increase in the autophagic clearance. Nevertheless, the results presented are not sufficient as to state that autophagy is not only induced but also active/functional in T2D patients.

The expression levels of autophagy-related parameters are significantly decreased in leukocytes of T2D patients under SS-31 therapy. In contrast, p62 protein expression, which is involved in aggresome formation and is itself degraded through autophagy, was increased in leukocytes from T2D patients by addition of SS-31. Of note, this was not due to changes in the gene expression of SQSTM1/p62 suggesting rather a SS-31 effect on autophagy. Our results support the existence of cross-talk between oxidative stress and autophagy in T2D [56], as SS-31 treatment of leukocytes of T2D patients reduces mitochondrial ROS production, which seems to prevent the increase induced by autophagic biomarkers. The specificity of this effect was shown by the fact SS-31 lacked the capacity to prevent the autophagic process induced by the pharmacological inducer of autophagy, rapamycin. As shown in many reports, rapamycin does not increase intracellular ROS levels (or can even diminish them) which is in keeping with our conclusion of SS-31 interfering with the autophagy observed in leukocytes from T2D patients through its capacity to scavenge mitochondrial ROS.

A link between ER stress, ROS production and autophagy could also be established considering the implication of cardiolipin in mitochondrial function including calcium buffering and mitophagy [57,58,59,60]. Given that in this work intracellular calcium levels in leukocytes from diabetics are enhanced concomitantly with increased presence of total and mitochondrial ROS, we could speculate that cardiolipin might be altered. With this and considering that SS-31 is a ROS scavenger that binds cardiolipin, we can speculate that cardiolipin may be involved in the effects exerted by SS-31. It could act as a regulator of mitophagy, explaining the reducing effect on autophagy seen in our work and could also affect calcium handling by mitochondria. It is widely known that calcium levels influence leukocyte function [61,62] and this occurs through NLRP3 signaling and the regulation of calmodulins and GTPases which participate in crucial processes in leukocytes such as innate defense and transmigration. Both aspects could reinforce SS-31 as a mitoprotective molecule that prevents leukocyte dysfunction. The effect of SS-31 on cardiolipin in this model and its relation to mitophagy seems a promising idea that needs to be explored in future studies.

It is important to mention that a possible limitation of this study are the potential interactions, synergisms, or detriments that may arise when studying or implementing novel drugs like SS-31 in a background affected by other medications such as statins. In this sense, previous research has stated that statins can have both detrimental [63,64,65,66] and beneficial effects [67,68,69,70,71,72,73,74,75,76,77], often unrelated to their lipid-lowering effect and rather associated with their pleiotropic actions. The variation of the effect is explained by the type of statin, the dose, the combination with other treatments and the experimental model. However, to our knowledge, there are no reports about the interference of statins with SS-31 when applied in combination, in patients or in animal studies.

## 5. Conclusions

In summary, our findings reveal a potential protective effect of novel SS-31 therapy in diseases with increased oxidative and an ER stress state such as T2D. It is important to highlight that mitochondrial accumulation of SS-peptides does not depend on alterations of ΔΨ_m_, which represents a major advantage to respect to other antioxidants [78,79,80]. The discovery of novel potential therapeutic strategies based on mitochondrial biology is key to future treatments, but further research is essential. The SS-31 peptide in particular represents a possible approach, through targeted delivery of antioxidants to mitochondria. In fact, in the present study we have demonstrated that SS-31 reduces ROS and could modulate ER stress and autophagy, key molecular pathways in cellular homeostasis, suggesting that this compound may exert beneficial effects that can be channeled for the treatment of T2D. Further investigations including clinical trials are required to elucidate these and other important mechanisms underlying the actions of SS-31 in treatment of T2D.

## Figures and Tables

**Table 1:** Protocol details and primer sequences.

**qRT-PCR Protocol**				
Temperature	95 °C	95 °C	60 °C	Melting
Time	10 min	10 s	30 s	Curve
No. of Cycles	1	40		
**Primers**				
**Target**	**Direction**	**Sequence (5′–3′)**		
*BECN1*	Forward	CCCCAGAACAGTATAACGGCA		
	Reverse	AGACTGTGTTGCTGCTCCAT		
*GRP78*	Forward	AAGAACCAGCTCACCTCCAACCC		
	Reverse	TTCAACCACCTTGAACGGCAA		
*DDIT3/CHOP*	Forward	AGAACCAGGAAACGGAAACAGA		
	Reverse	TCTCCTTCATGCGCTGCTTT		
*GAPDH*	Forward	CGCATCTTCTTTTGCGTCG		
	Reverse	TTGAGGTCAATGAAGGGGTCA		
*SQSTM/P62*	Forward	GATTCGCCGCTTCAGCTTCTG		
	Reverse	CTGGAAAAGGCAACCAAGTCC		

**Table 2:** Anthropometric and analytical parameters.

	Control	Type 2 Diabetes	*p*-Value	BMI-Adjusted *p*-Value
*N*	53	61	-	-
Male (%)	47.2	52.5	ns	ns
Age (years)	51.7 ± 9.3	55.1 ± 10.2	ns	ns
Weight (Kg)	72.9 ± 18.8	85.6 ± 15.5	*p* < 0.001	*p* < 0.001
BMI (kg/m^2^)	25.8 ± 5.4	31.4 ± 5.6	*p* < 0.001	-
Waist circumference (cm)	85.8 ± 13.2	104.0 ± 11.9	*p* < 0.001	*p* < 0.01
SBP (mmHg)	23.3 ± 19.7	145.8 ± 14.8	*p* < 0.001	*p* < 0.001
DBP (mmHg)	73.6 ± 10.9	74.2 ± 25.6	ns	ns
Glucose (mg/dL)	95.6 ± 13.6	154.0 ± 49.8	*p* < 0.001	*p* < 0.001
Insulin (µUI/mL)	7.56 ± 3.55	16.27 ± 9.09	*p* < 0.001	*p* < 0.01
HOMA-IR	1.71 ± 0.95	6.23 ± 4.64	*p* < 0.001	*p* < 0.001
HbA1c (%)	5.32 ± 0.36	7.42 ± 1.57	*p* < 0.001	*p* < 0.001
Total cholesterol (mg/dL)	198.8 ± 35.5	168.0 ± 37.7	*p* < 0.001	*p* < 0.001
HDL-c (mg/dL)	57.3 ± 19.9	43.1 ± 9.2	*p* < 0.001	*p* < 0.001
LDL-c (mg/dL)	122.1 ± 28.9	93.7 ± 30.6	*p* < 0.001	*p* < 0.001
Triglycerides (mg/dL)	93.0 (26.5–150.5)	133.0 (94.0–170.0)	*p* < 0.01	*p* < 0.01
hs-CRP (mg/L)	1.17 (0.46–2.40)	2.92 (1.88–6.39)	*p* < 0.001	*p* < 0.001

Data are shown as mean ± SD and were compared by a Student’s *t* test for parametric variables, while they are shown as median and were compared by a Mann–Whitney *U* test (25th and 75th percentiles) for non-parametric variables. A univariate general linear model was used to adjust changes for BMI. A Chi-Square test was used to compare proportions among groups. ns: not significant.

## Supplementary Materials

The following are available online at https://www.mdpi.com/2077-0383/8/9/1322/s1, Figure S1: Leukocyte-enfothelium interaction evaluation under SS-31 and SS-20 treatment.
