# Supplementary material for: The Mitochondrial Antioxidant SS-31 Modulates Oxidative Stress, Endoplasmic Reticulum Stress, and Autophagy in Type 2 Diabetes
Source: J Clin Med. 2019 Aug 28;8(9):1322. doi: 10.3390/jcm8091322 (PMC6780723; doi:10.3390/jcm8091322)
Supplement: Supplementary file 1 [file jcm-08-01322-s001.pdf]

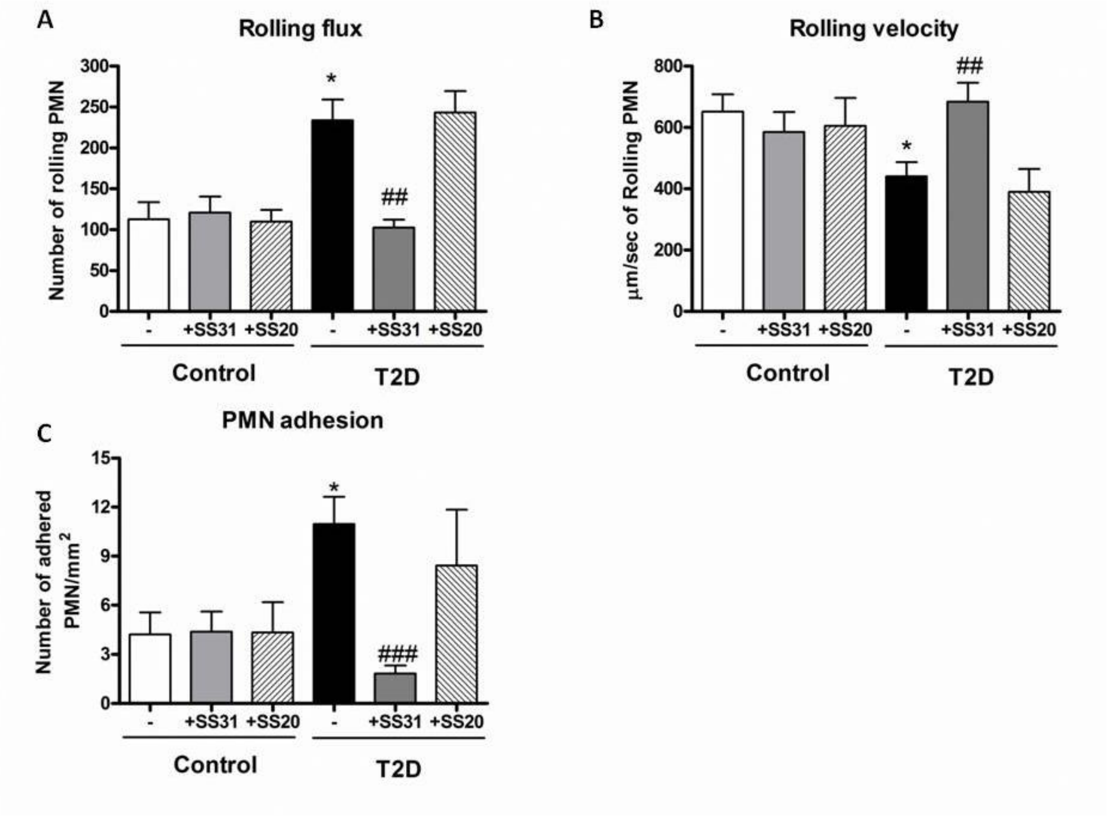

Figure S1. Leukocyte-enfothelium interaction evaluation under SS-31 and SS-20 treatment. (**A**) Number of rolling PMN in 1 min, (**B**) velocity of this rolling PMN and (**C**) PMN adhesion to the endothelial monolayer. * $p < 0.05$ with regard to control group; ## $p < 0.01$ ### $p < 0.001$ vs. non-treated T2D group.
